# Improvement of thermal-stability of chondroitinase ABCI immobilized on graphene oxide for the repair of spinal cord injury

**DOI:** 10.1038/s41598-023-45555-9

**Published:** 2023-10-25

**Authors:** Atefeh Hassanli, Sara Daneshjou, Bahareh Dabirmanesh, Khosro Khajeh

**Affiliations:** 1 Department of Nanobiotechnology, Faculty of Biological Science, Tarbiat Modares University, P.O. Box 14115-175, Tehran, Iran; 2 Department of Biochemistry, Faculty of Biological Science, Tarbiat Modares University, Tehran, Iran

**Keywords:** Biotechnology, Biochemistry, Enzymes

## Abstract

Spinal cord injury healing has been shown to be aided by chondroitinase ABC I (cABCI) treatment. The transport of cABCI to target tissues is complicated by the enzyme's thermal instability; however, cABCI may be immobilized on nanosheets to boost stability and improve delivery efficiency. This investigation's goal was to assess the immobilization of cABC I on graphene oxide (GO). for this purpose, GO was produced from graphene using a modified version of Hummer’s process. the immobilization of cABC I on GO was examined using SEM, XRD, and FTIR. The enzymatic activity of cABC I was evaluated in relation to substrate concentration. The enzyme was then surface-adsorption immobilized on GO, and its thermal stability was examined. As compared to the free enzyme, the results showed that the immobilized enzyme had a greater Km and a lower Vmax value. The stability of the enzyme was greatly improved by immobilization at 20, 4, 25, and 37 °C. For example, at 37 °C, the free enzyme retained 5% of its activity after 100 min, while the immobilized one retained 30% of its initial activity. The results showed, As a suitable surface for immobilizing cABC I, GO nano sheets boost the enzyme's stability, improving its capability to support axonal regeneration after CNC damage and guard against fast degradation.

## Introduction

The enzyme known as chondroitinase ABC (cABC; EC 4.2.2.4) is responsible for cleaving the chondroitin sulfate’s 1,4-glycosidic bond. *P. vulgaris* has yielded two isoforms, cABC I and cABC II, which have been recombinantly produced in *Escherichia coli*^1^. Both isoforms may break down members of the glycosaminoglycan family, such as chondroitin sulfate, dermatan sulfate, and chondroitin 4-sulfate, however cABC I has a much higher catalytic efficiency than cABC II due to its 997 amino acids and 112.5 kDa molecular mass^2^. An injury to the central nervous system (CNS), particularly to the spinal cord, results in permanent tissue damage that activates glial cells at the location of the lesion and creates a glial scar^3^. Proteoglycans, especially chondroitin sulfate proteoglycans, which serve as a considerable barrier to regenerating axons, make up the majority of the glial scar^4,5^. The cleavage of chondroitin sulfate proteoglycan chains by the cABC enzyme opens up a possible therapeutic avenue for the treatment of spinal cord injuries and nerve regeneration. In animal models of CNS damage, therapy with cABC improves motor and sensory function, promotes neuronal connection regeneration, and plasticity, according to experimental findings^6,7^. The sustained delivery of bioactive cABC is a serious challenge, requiring highly invasive techniques or the implantation of delivery systems directly into the tissue and is a serious obstacle to the practical use of cABC for spinal cord therapy^8^. cABC has been shown to be a highly labile enzyme that inactivates after few days in solution^9^, although weeks after the damage, proteoglycan deposition and the development of the glial scar persist. As the activity of cABC also quickly declines at 37 °C, it is required to increase the thermal stability of cABC^2^. Otherwise, repeated injections over a period of days to weeks or local infusions may result in infection and a body immune system reaction. To boost the thermal stability and activity of cABC, a variety of techniques have been employed, including the use of stabilizing chemicals, mutagenesis, chemical modification, and immobilization. According to reports, glycerol, sorbitol, and trehalose all help to stabilize cABCI^10^. Cosolvents also slow down the rate of oxidative and proteolytic inactivation of cABCI^11^. Moreover, the Proteus vulgaris cABCI's activity, stability, and tertiary structure were examined in glycerol- and betaine-based deep eutectic solvents. The results demonstrated that a glycerol and betaine or choline combination might improve the stability and activity of the enzyme^12^. In cABCI, mutagenesis is crucial for maintaining the stability of the enzyme. In this manner, the enzyme is deactivated and rendered unstable by the substitution of His475 with Ala and Try476 with His and Ala^13^. The immobilization technique has become a common way to stabilize the enzyme since the enzyme attachment to a carrier limits the unfolding of the enzyme^14,15^. At different temperatures (20, 4, 25 and 37 °C), immobilizing cABCI by trapping it within porous silicon nanoparticles has been shown to considerably boost enzyme stability^16^. Moreover, cABCI has been immobilized using magnetite nanoparticles, and the findings indicated that the immobilized cABCI's storage stability at low temperatures was greatly improved^17^. However, due to potential issues with the nanoparticle crossing the structural complexity of the CNS and the blood-spinal cord barrier, careful selection of a specific nanoparticle type is a critical issue. Due to its outstanding qualities, including controllable form and size, cheap cost, and relatively good biocompatibility, graphene derivatives have drawn a lot of attention and research interest in this area^18,19^.Monolayer graphene, ultrathin graphite, few-layer graphene, graphene nano sheets, and graphene or graphene oxide (GO) are examples of graphene derivatives. GO is a polar derivative of graphene that is water soluble, and its biocompatibility, large specific surface area, excellent solubility, and low toxicity permits extensive usage in biology and medicine, and it is a suitable host for enzyme immobilization^20^. Via van der Waals, ionic, and—stacking interactions, the many oxygen-containing groups on GO promote enzyme attachment^21^. It has been shown that enzyme immobilization on GO sheets is possible without the need for further surface modification or cross-linking agents^22^. Successful lipase immobilization has also been accomplished on the magnetically separated Fe_3_O_4_/GO surface^23^. Although there have been many advances in enzyme immobilization, designing an immobilization platform for biocatalysis involving nanoparticles that can immobilize the cABCI and enhance its stability and activity is still difficult due to the protein damage caused by the frequently "harsh" reaction conditions required for these reactions^24,25^. It has been reported that employing the graphene oxide in scaffold compounds can play a role in regeneration of neuronal tissues^26^. Also, considering the role of the cABCI enzyme in the neuronal connection regeneration, the immobilization of this enzyme on graphene oxide can be important in the clinical application.

As far as we are aware, there has never been a report on the use of GO as a nanocarrier for cABCI. As a result, in the current work, GO was produced utilizing a modified version of Hummer's approach and cABC I enzyme was immobilized on GO through direct (electrostatic) interaction. Finally, the kinetic parameters and thermal stability of the free and immobilized enzyme were investigated.

## Materials and methods

### Materials

From Sigma-Aldrich, we obtained graphite powder, H_2_SO_4_, NaNO_3_, NaOH, H_2_O_2_ (30%), K_3_Fe(CN)_6_, KMnO_4_, and chondroitin 4-sulfate (USA). All of the chemicals were analytical reagent grades and were used directly out of the package. In this investigation, Ni–NTA agarose from Qiagen in the United States and Isopropyl-β-d-thiogalactopyranoside (IPTG) from Takara in Japan were both utilized. The studies employed substances with an analytical purity grade.

### Synthesis of GO

GO was created with a modified version of Hummer's approach and was based on earlier research^27,28^. For this purpose, 0.5 g of natural graphite was dissolved in 23 mL of sulfuric acid and 0.5 g of sodium nitrate in an ice bath while being constantly stirred. In order to avoid explosion and overheating, potassium permanganate (3.0 g) was progressively added to the acid mixture while stirring and maintaining the temperature below 20 °C. After 12 h of stirring at 35 °C, the liquid was diluted by adding 500 cc of water while vigorously swirling. The suspension was then treated with a 30% H_2_O_2_ solution to verify that the reaction with the KMnO_4_ was complete (5 ml). After filtering, drying, and washing the resultant mixture with HCl and water, respectively, graphene oxide sheets were produced.

### Expression and purification of cABC I

In BL21 *E. coli* host cells, cABC I was expressed using the same technique as described by Daneshjou et al.^24,25^. By using SDS-PAGE analysis, the purity and molecular mass were evaluated. The Bradford technique was used to quantify the protein content at 595 nm using bovine serum albumin as the standard^29^.

### Immobilization of cABC I on GO

cABC I enzyme solution (concentration of 2.5 mg/mL) was applied to 0.04 mg/ml of GO in order to physically adsorb the enzyme onto the substance. After that, the mixture was put in a sonication bath (made by JAMGS, model SONLC 600 M) to uniformly and thoroughly disperse the nanoparticles throughout the enzyme solution. The resultant solution was centrifuged at 15,000×*g* for 30 min after being incubated on the stirrer at 4 °C for 12 h and being rinsed three times with phosphate buffer. The pellet resuspension and the evaluation of enzyme stability and activity both employed the phosphate buffer.

### Enzymatic activity assay

The test for enzymatic activity was carried out in accordance with other investigations^2,11^. By measuring the product spectrophotometrically at 232 nm in 50 mM phosphate buffer (pH 6.8) at 25 °C, the activity of cABC I was investigated. In a nutshell, 20 μl of pure enzyme were mixed with 290 μl solution containing various concentrations of chondroitin-4-sulfate in 50 mM phosphate buffer. Chondroitin-4-sulfate (C4S) was the substrate. The product's molar extinction coefficient (ε), which is 3800 M^−1^ cm^−1^, was utilized to calculate the activity. A unit (U) of cABC I activity is the amount of the enzyme required to convert 1 μmol of chondroitin-4-sulfate into unsaturated disaccharides per minute under the test conditions. Using the Prism version 5.0 software (La Jolla, CA, USA) nonlinear regression function, data were fitted to the Michaelis–Menten equation. The standard deviations were all within 5% after three runs of each experiment.

### Stability

cABCI lyase stability was analyzed by incubating the free and immobilized enzyme at − 20, 4, 25 and 37 °C for 3 h, and after various time intervals, the samples were assayed for their residual activity at 25 °C as mentioned above. The experiments were carried out at least in triplicate and the standard deviations has been reported as ± 5%.

### Support characterization

An image of a GO was obtained by atomic force microscopy (AFM) (Autoprobe CP microscopy). Scanning electron microscopy (SEM), using a Philips XL30 scanning electron microscope, was used to study the morphology of GO and immobilized cABC I. (Netherlands). GO's crystalline makeup and phase purity were examined Using a Panalytical X Pert Pro XRD with Cu Ka 1.5406 A° radiation. The X-ray diffractometer was used to implement the XRD analysis. Raman spectroscopy was performed on GO using an XloRA Plus device (Horiba, Japan). GO's molecular structure was studied using an Fourier Transform Infrared Spectroscopy (FTIR) spectrometer both before and after cABC I immobilization (Varian Inc. 7000e).

## Results and discussion

### Expression and purification of cABC I

**Figure 1:**
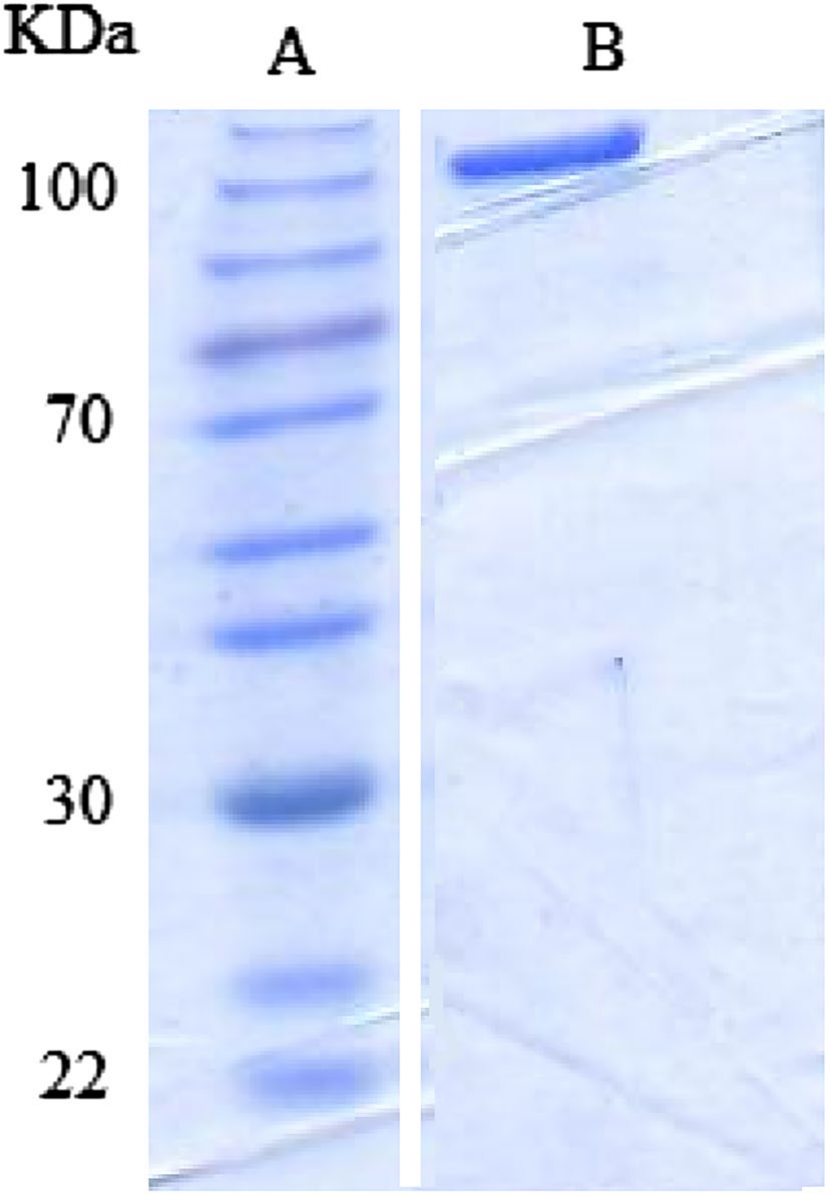
SDS-PAGE analysis of protein ladder (A) and purification of cABC I at expected molar mass of 112 kDa (B).

In the current investigation, an N-terminal 6 × His tag was used to express cABC I in BL21 *E. coli* host cells. SDS-PAGE analysis determined the molecular mass and purity of cABC I, showing (Fig. 1A,B). cABC I has a molecular mass of 112.5 kDa and 997 amino acids, according to a prior research^2^. The Bradford technique was used to quantify the protein content at 595 nm using bovine serum albumin (BSA) as the standard.

### Chondroitinase immobilization on GO

**Figure 2:**
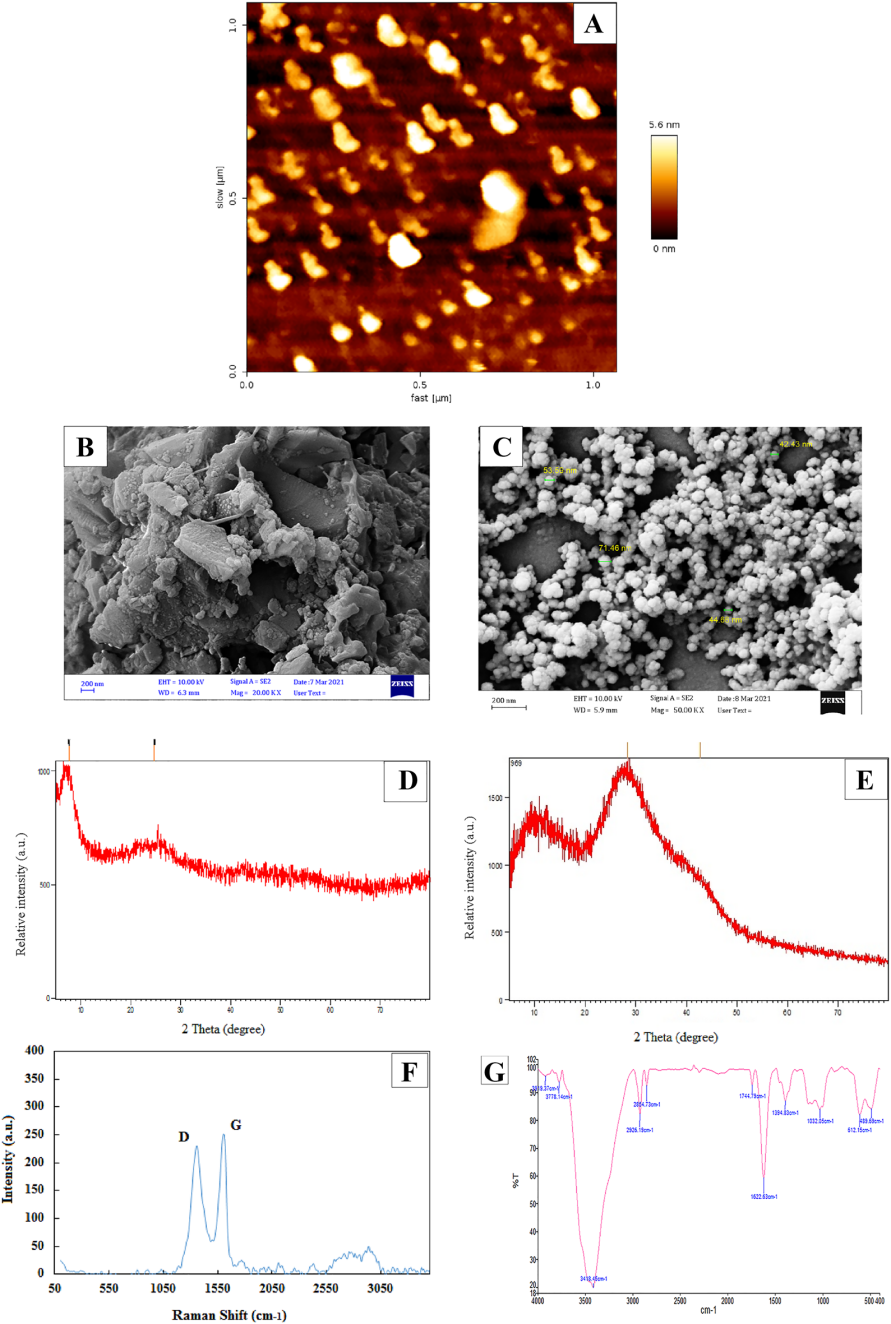

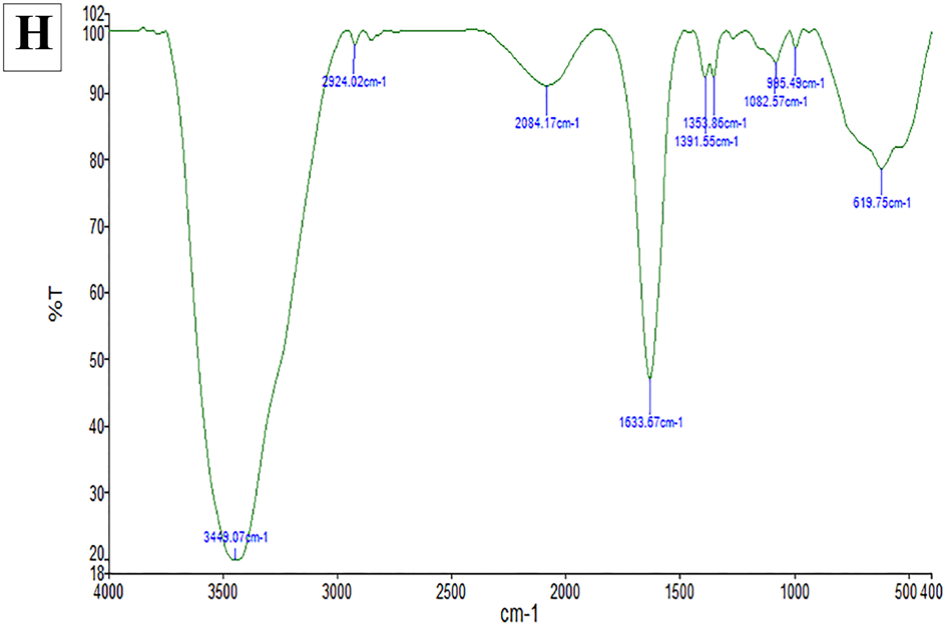
The AFM and SEM of graphene oxide (GO) (**A**,**B**) and immobilized cABC I on GO (**C**), XRD spectrum of GO (**D**)and XRD spectrum of immobilized cABCI on GO (**E**), Raman spectra of graphene oxide (**F**), FT-IR spectrum of pure GO (**G**) and FT-IR spectrum of immobilized cABC I on GO (**H**).

The enzyme was immobilized using a nano substrate made of graphene oxide powder. First, the three-dimensional structure of the enzyme was examined using the YASARA bioinformatics tool (Version 20.12.24). Due to the abundance of aspartate and glutamate in the enzyme structure, it was predicted that the ions in graphene oxide are responsible for trapping the carboxylic acid groups from aspartate residues (Asp and glutamate (Glu)) in the side chains of amino acids. The immobilization procedure thus occurred by using the strategy of direct contact of chondroitinase enzyme with GO. It is worth noting that, Enzyme was immobilized on graphene oxide using electrostatic interactions. Zeta analysis shown that graphene oxide has a negatively charge (− 23.9), which was consistent with the studies of Cao et al.^30,31^, while the enzyme with a pI of 8.5 is positively charged^16^. Therefore, interaction can be visualized as an electrostatic attraction. Eventually, GO was added to the enzyme in phosphate buffer (pH6.8) and swirled for 12 h at 4 °C. To better balance the relationship between enzyme loading and the activity of the immobilized enzyme, a number of enzyme concentrations were examined for their effectiveness in imprisoning within the GO surface. The activity initially increased as the enzyme concentration rose; but, at concentrations greater than 0.2 mg/mL (where about 40 g of enzyme were loaded per mg of GO), the activity remained constant. As seen in Fig. 2B, it seems that the enzyme's extra layers smoothed down the surface^16^.

### Free and immobilized chondroitinase characterization

#### AFM and SEM results

Atomic force microscopy (AFM) and scanning electron microscope (SEM) images of graphene oxide (GO) samples are shown in Fig. 2A and B, respectively. Figure 2A shows a three-dimensional representation of the surface in which the graphene oxide surface is drawn with good resolution accuracy and by collecting accurate data and converting them into three-dimensional models. The heights are reported in all three axes of length, width and height. Figure 2B,C shows SEM micrographs of synthetic GO and cABC I immobilized on GO. From these images, it is clear that GO has a layered structure that allows for the production of ultrathin and homogenous graphene sheets. These films may be folded or continuous at times, and the edges of separate sheets, including kinked and wrinkled sections, can be seen, These images are almost consistent with previous studies^32–34^. Alam et al. found GO lamellar structures in SEM micrographs^35^. SEM analysis revealed that the graphene nano sheets had thin aggregates that were uniformly distributed and had smaller pores. The GO sheets hung straight and did not budge. SEM images of immobilized cABC I on GO (Fig. 2B,C) demonstrate that the enzyme molecules were effectively immobilized on GO and that a layer of the enzyme had coated the surface of graphene oxide following immobilization. According to the results of this research, many experimental investigations have looked into the immobilizing potential of nanoparticles and nanocomposites on enzymes. It has been reported that pancreatic lipase enzyme may be immobilized by three-dimensional magnetic graphene oxide-magnetite polyvinyl alcohol nanocomposites^36^. The capacity of carboxyl-functionalized graphene oxide to effectively immobilize the lipase enzyme for catalysis in organic solvent has also been shown^37^.

#### X-ray diffraction (XRD) results

The XRD spectrum of graphene oxide in Fig. 2D shows diffraction peak before 10◦. The proper orientation of the graphene oxide base sheets is in accordance with those reported by Marcano et al.^38–40^. Another diffraction peak observed at 25°,indicates the distance between the graphene layers, which is very consistent with the reported in^41^ and shows the good order of graphene interlayer spacing^34,40^. The diffraction peak at 25° disappears completely when GO is dried^39,42^. These phenomenan are similar to our results reported in the research of Yan et al. and Khakpour et al., in which for GO synthesized by the Hummers method, the reemergence of diffraction lines is C(002) at 25°, revealing the typical structure of graphite and multiple layers of graphene^43^ as well as the polycrystalline nature of graphene that has also been reported in a recent study^44^. The XRD spectrum of immobilized cABC I on GO is seen in Fig. 2E. No additional impurity phase was seen in XRD, and the typical peaks for immobilized cABC I on GO correspond well with 42, 30 (JCPDS card). Also, the XRD data demonstrated that the chondroitinase ABC-I enzyme's adsorption on graphene oxide somewhat altered the peak's position and intensity in comparison to free graphene oxide, confirming the enzyme's physical attachment to the material. XRD were used to characterize synthesized GO, which is consistent with other investigations^43,45^.

#### FTIR and Raman results

Raman spectroscopy was utilized to confirm the GO synthesis (Fig. 2F). As can be seen from this figure, the main peaks for GO were observed around 1350 cm^−1^ (D band), 1590 cm^−1^ (G band), which ensure lattice distortions and 2690 cm^−1^ (2D band) are related to the set of graphene layers^38,46^. in the study of Strankowski et al. performed on GO, the most important signals can be seen as strong D and G bands^47,48^. The intensity of the D band depends on the number of oxygen atoms on the GO surface^49^. The results indicate that the synthesis of graphene oxide is consistent with the studies conducted on graphene oxide^50,51^. FTIR measurements of graphene oxide (GO) and immobilized cABC I on GO were conducted to confirm the immobilization of cABC I on GO (Fig. 2G and H). In addition to a distinct wide peak between 3100 and 3700 cm^−1^, GO also showed discernible peaks at 1744 cm^−1^, 1622 cm^−1^, 1394 cm^−1^, and 1032 cm^−1^, which corresponded to the carboxylic acid groups' O–H and C=O stretching, aromatic C–C stretching, O–H deformation, and the C–O, respectively (Fig. 2G). Absorption peaks seen at 1622 cm^−1^ and 612 cm^−1^ may be ascribed to the stretching vibration of C=C and C=O of carboxylic acid and carbonyl groups located at the margins of graphene oxide. The absorption peak at 2926 cm^−1^ and 2850 cm^−1^ represents the symmetric and anti-symmetric stretching vibrations of CH_2_. The stretching vibrations of the carbon atoms in carboxylic acid and alcohol, respectively, are what cause the absorption maxima at 1394 cm^−1^ and 1032 cm^−1^, respectively. These oxygen-containing groups show that the graphite has undergone oxidation^38,52^. Because of the polar groups in its structure, GO is a hydrophilic substrate because they cause hydrogen bonds to form between the molecules of graphite and water^25^ separated graphene sheets that have been covered with oxygen functional groups on both the basal planes and the edges, formed the physico-chemical structure of GO^53–55^. The ionization of carboxyl groups in graphene oxide, which transforms it into a potent polar molecule and stabilizes the ensuing graphite dispersions, causes colloidal dispersions of graphene oxide in solvents including water, alcohol, and organic solvents^56,57^. Similar to what was previously seen in the stabilization of cholesterol oxidase enzyme on the substrate of graphene oxide nanosheets or polyphenol oxidase on the same substrate^58,59^, comparison of IR spectra before and after cABC I immobilization revealed that cABC I adsorption on GO significantly changed the location and intensity of the peaks (Fig. 2H). The results obtained from FTIR spectrometry are in agreement with reports on the FTIR spectrum of Hammers' graphene oxide in other studies^44,45,60,61^.

### Kinetic studies of free and immobilized cABCI

**Figure 3:**
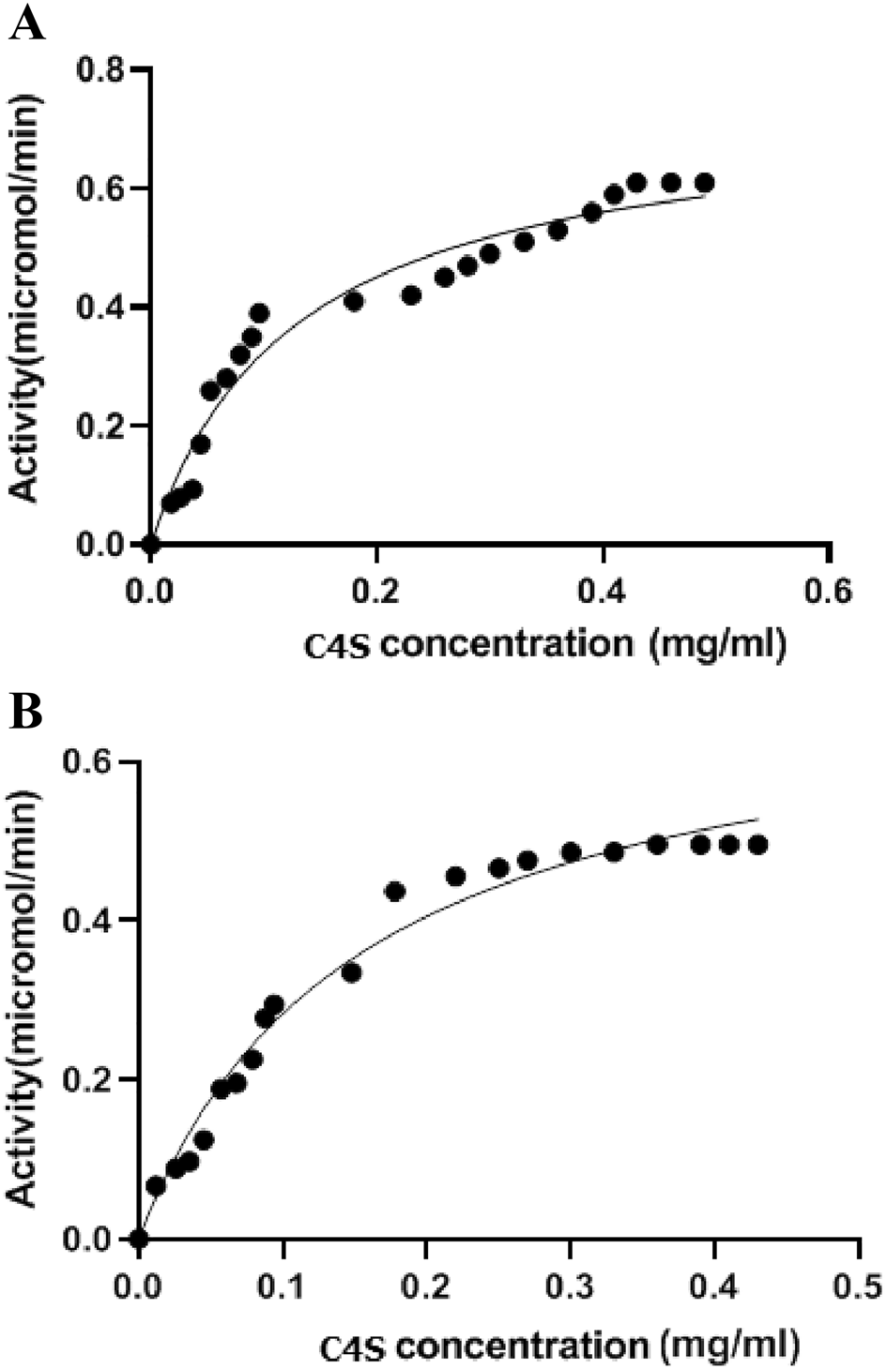
Kinetic analysis of chondroitinase ABC I. The initial reaction rates were plotted versus various substrate (C4S) concentrations for chondroitinase ABC I. (**A**) Free enzyme, (**B**) Immobilized enzyme on GO. Using Prism. Standard deviations were within 5% of the experimental values.

The kinetic parameters for free and immobilized enzymes were derived from the Michaelis–Menten plot after the initial reaction rates were plotted against various substrate concentrations (Fig. 3). For immobilized and free enzyme, the Km values were around 0.15 mg/mL and 0.12 mg/mL, respectively (Table 1). For the immobilized enzyme, the values of Michaelis constant (Km), (maximum effective velocity)Vmax, and specific activity values were about 0.15 mg/mL, 0.49 mol min^−1^, and 30 mol min^−1^ mg^−1^. The free enzyme's Km value was marginally lower than the immobilized enzyme's (Table 1), indicating a decreased affinity for substrate that may be brought on by substrate diffusion onto the GO surface^62^. We further hypothesize that the conformational changes may be to blame for the decreased affinity towards the substrate after immobilization. Noting that the immobilization method does not control the appropriate orientation of the immobilized enzyme on the support, some of the active sites are lost and the specific activity is reduced in comparison to the free enzyme. The loss of part of the immobilized enzyme's active sites owing to the varied orientations on the GO surface may be the cause of the Vmax and specific activity of the immobilized enzyme decreasing compared to free enzyme^16,27,63^. is constrained, the enzyme's ability to change the substrate will be hindered, which will lower the rate of product production as a function of enzyme concentration^16,53^. It is worth mentioning, several studies have shown that when nanoparticles enter the body, interact with biological fluids, such as plasma and form protein corona, which can lead to different biological results^64,65^. For example, in a study conducted by Akhavan et al.^64^, it was found that GO can cause different biological responses in the presence of coronas obtained from different types of disease. As a result, it is possible that the activity of the immobilized enzyme in the body's cellular fluids undergoes changes under the influence of protein corona, which requires further studies.

**Table 1:** Kinetic parameters of the free and immobilized chondroitinase.

Sample	Km (mg/mL)	V_max_ (μmoles min^−1^)	Specific activity (μmoles min^−1^ mg^−1^
Free cABC I	0.12 ±0.005	0.72 ± 0.005	59 ± 0.005
Immobilized cABC I on graphene oxide	0.15 ± 0.005	0.49 ± 0.005	30± 0.005

### Thermal stability

**Figure 4:**
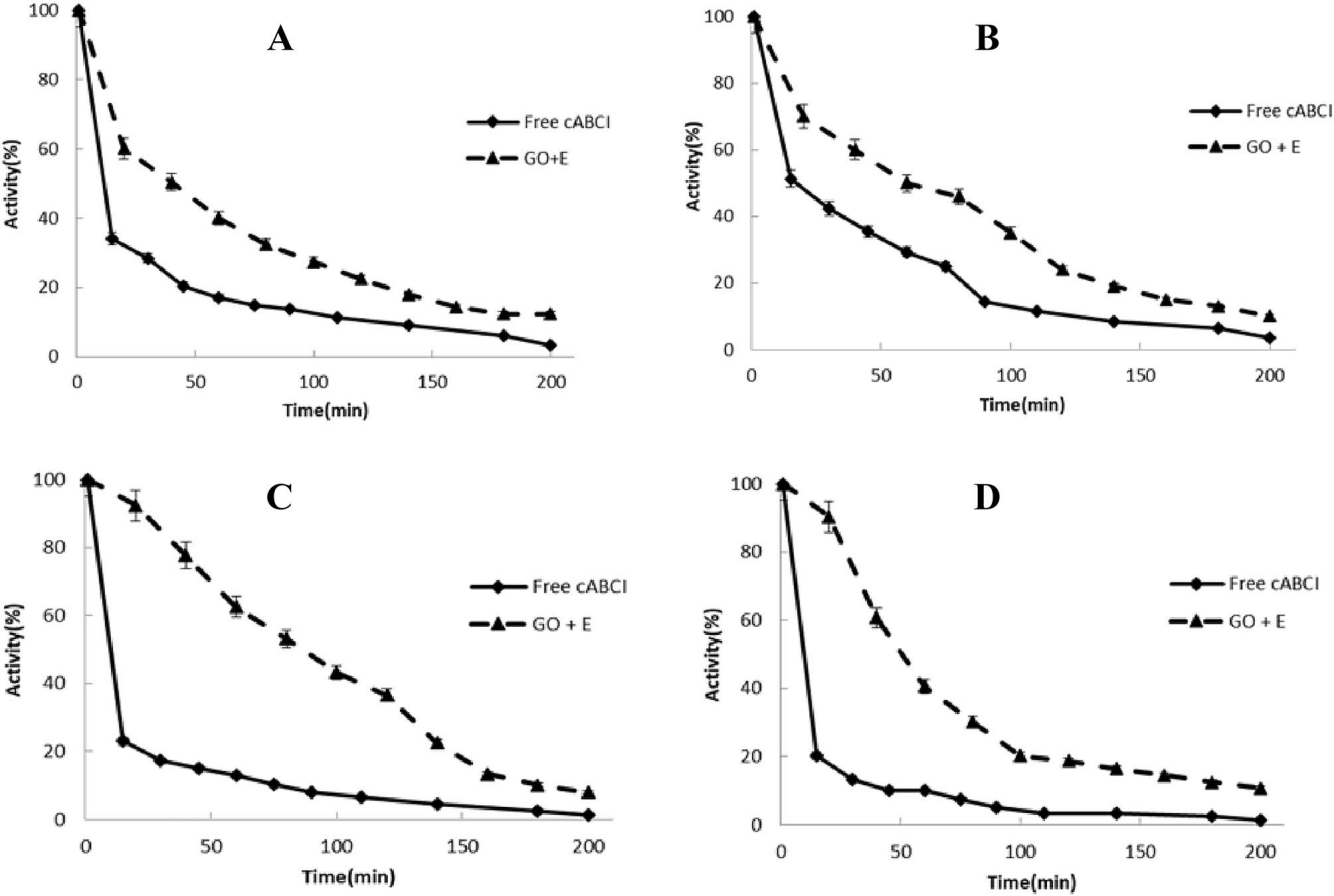
Thermal stability of immobilized enzyme on GO (– –) and free enzyme (–) at (**A**) − 20 °C (**B**) 4 °C (**C**) 25 °C (**D**) 37 °C in 50 mM phosphate buffer (pH 6.8). Standard deviations were within 5% of the experimental values.

After enzyme immobilization, some characteristics of the enzyme molecule, such as its catalytic activity or thermal stability, differ from those of its counterpart in solution^24^. These alterations are most likely the result of modifications to the immobilized enzyme's intrinsic activity or of interactions between the immobilized enzyme and the substrate in a microenvironment distinct from the bulk solution. Hence, the stability of free and immobilized chondroitinase on the GO was investigated at − 20, 4, 25 and 37 °C. The purified chondroitinase after Ni–NTA column was previously reported to preserve 80% of its activity in 300 mM imidazole after 21 days kept at 4 °C, but the enzyme had no activity after 3 days at 20 °C and 17 days at 25 °C^7,11,16^. Imidazole concentration was dropped to 10–15 mM after purification using Centricon to test the thermal stability since there was no activity after dialysis in the absence of the compound. Thermal stability was next evaluated using free (in 10–15 mM imidazole) and immobilized enzyme (no imidazole). At all temperatures studied, the immobilized chondroitinase on GO became more stable in contrast to the free enzyme (Fig. 4A–D). The results showed that at all tested temperatures, the immobilized chondroitinase was more stable than its free counterpart. Another thing to keep in mind is that while activity was reduced in this immobilized system over a short period of time, it did so more gradually. For instance, at 4 and 37 °C, the immobilized enzyme maintained around 60% and 50% of its original activity after 50 min, respectively, but the activity of the free enzyme reached 30% at 4 °C and 10% at 37 °C and practically lost its activity after 100 min (Fig. 4A–D). It's important to note that over the course of one month, the amount of immobilized enzyme activity stays essentially constant at all temperatures. But, from the comparison of the results with the previous studies (Daneshjou et al.^16,24^), it can be concluded that, at 4, 25 and 37 °C, the inactivity rate of immobilized enzyme on GO is slower than immobilized enzyme on porous silicon. In addition, the importance of employing GO in this study is because Graphite Oxide is more biocompatible and more stable than porous silicon. The porous silicon structure is unstable and disintegrates faster than graphene oxide. As a result, graphite oxide is a better option for drug carriers. In general, graphene oxide has a long biodegradation period and may be kept longer with continuous drug release after injection. As a result, drug release can be controlled for a long time, and complications from repeated injections and side effects from high doses can be avoided. It was previously reported that, lipase and dehydrogenase were successfully stabilized by the immobilization on GO^54^.

In addition to the afromentioned advantages, it has been widely proven that GO plays a very important role in the proliferation and differentiation of neural cells^66,67^. Although Chondroitinase enzyme is also employed for the treatment of spinal lesions, it has low thermal stability and for therefore, it requires repeated injections in the lesion site. However, by immobilizing this enzyme on the GO substrate, the stability of this functional enzyme has increased. Considering the substrate (GO) itself plays a role in the proliferation of neural cells, a promising approach to repair spinal cord injuries can be introduced in the future.

## Conclusion

GO was successfully synthesized in the current work utilizing a modified version of Hummer's technique. XRD, FTIR and SEM were used to characterized graphene oxide and enzyme immobilization. We have shown that immobilized cABC I activity on GO followed Michaelis–Menten kinetics, and had lower Vmax and Higher Km than free enzyme. The findings also indicated that graphene oxide can work as a heat-labile chondroitinase carrier. Because after immobilization on graphene oxide, the immobilized enzyme was considerably more stable (at all tested temperatures). As a result, the GO could be an effective stabilizing medication delivery system. While further experimental testing is required, the findings of this study are highly encouraging and provide an intriguing strategy for future spinal cord injury recovery (Supplementary Information).

### Supplementary Information

Supplementary Information.

## Data Availability

The datasets used and/or analysed during the current study available from the corresponding author on reasonable request.
