# Supplementary material for: Improvement of thermal-stability of chondroitinase ABCI immobilized on graphene oxide for the repair of spinal cord injury
Source: Sci Rep. 2023 Oct 25;13:18220. doi: 10.1038/s41598-023-45555-9 (PMC10600109; doi:10.1038/s41598-023-45555-9)
Supplement: Supplementary file 1 — Supplementary Information. [file 41598_2023_45555_MOESM1_ESM.pdf]

.

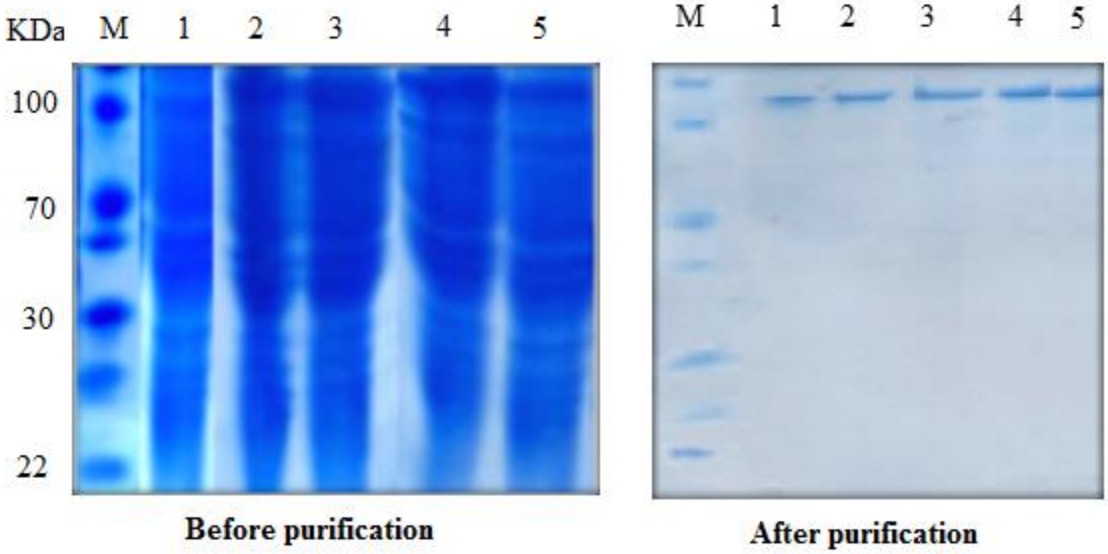

M: Marker or ladder, Numbers 1-5:Number of repetitions

Complementary picture for fig.1: full length membranes.
